# A phase I study of dexosome immunotherapy in patients with advanced non-small cell lung cancer

**DOI:** 10.1186/1479-5876-3-9

**Published:** 2005-02-21

**Authors:** Michael A Morse, Jennifer Garst, Takuya Osada, Shubi Khan, Amy Hobeika, Timothy M Clay, Nancy Valente, Revati Shreeniwas, Mary Ann Sutton, Alain Delcayre, Di-Hwei Hsu, Jean-Bernard Le Pecq, H Kim Lyerly

**Affiliations:** 1 Department of Medicine, Duke University Medical Center, Durham, NC, USA; 2 Department of Surgery, Duke University of Medical Center, Durham, NC, USA; 3 Anosys Inc., Menlo Park, CA, USA; 4 Currently at Genentech, Inc., South San Francisco, CA, USA

## Abstract

**Background:**

There is a continued need to develop more effective cancer immunotherapy strategies. Exosomes, cell-derived lipid vesicles that express high levels of a narrow spectrum of cell proteins represent a novel platform for delivering high levels of antigen in conjunction with costimulatory molecules. We performed this study to test the safety, feasibility and efficacy of autologous dendritic cell (DC)-derived exosomes (DEX) loaded with the MAGE tumor antigens in patients with non-small cell lung cancer (NSCLC).

**Methods:**

This Phase I study enrolled HLA A2+ patients with pre-treated Stage IIIb (N = 4) and IV (N = 9) NSCLC with tumor expression of MAGE-A3 or A4. Patients underwent leukapheresis to generate DC from which DEX were produced and loaded with MAGE-A3, -A4, -A10, and MAGE-3DPO4 peptides. Patients received 4 doses of DEX at weekly intervals.

**Results:**

Thirteen patients were enrolled and 9 completed therapy. Three formulations of DEX were evaluated; all were well tolerated with only grade 1–2 adverse events related to the use of DEX (injection site reactions (N = 8), flu like illness (N = 1), and peripheral arm pain (N = 1)). The time from the first dose of DEX until disease progression was 30 to 429+ days. Three patients had disease progression before the first DEX dose. Survival of patients after the first DEX dose was 52–665+ days. DTH reactivity against MAGE peptides was detected in 3/9 patients. Immune responses were detected in patients as follows: MAGE-specific T cell responses in 1/3, increased NK lytic activity in 2/4.

**Conclusion:**

Production of the DEX vaccine was feasible and DEX therapy was well tolerated in patients with advanced NSCLC. Some patients experienced long term stability of disease and activation of immune effectors

## Introduction

Vaccine immunotherapy as an approach to cancer treatment has evolved over the last 10 years as the basic biology of the immune response has been elucidated. Tumor-associated antigens that are capable of eliciting cytotoxic T cell responses have been identified. Among the most frequently expressed across many malignancies are the MAGE antigens, originally described in melanoma, but expressed by other tumors including non-small cell lung cancer (NSCLC) [1-3]. Immune responses to MAGE 3 have been correlated with clinical outcome in melanoma patients [4]. This has lead to many tumor antigen-specific strategies for the treatment of cancer, including the use of an immunodominant peptide alone, protein or peptide-pulsed dendritic cells, and antigen/co-stimulatory fusion proteins expressed from viral vectors. Although each strategy has its proponents, none have achieved the goal of activating significant immune responses that correlate with clinical responses in a majority of patients; therefore, there is a continued need to develop even more effective strategies. Recently, a novel platform for delivering high levels of antigen in conjunction with costimulatory molecules has been described, called exosomes, cell-derived lipid vesicles that express high levels of a narrow spectrum of cell proteins.

A variety of cells have been shown to release exosomes including dendritic cells [5], B lymphocytes [6], T lymphocytes [7], mast cells [8], platelets [9], and tumor cells [10]. The small (60–90 mm) vesicles form within the late endosomes or multivesicular bodies and have biologic functions dependent on the cell type from which they were secreted [11-13]. Originally described as vesicles released from reticulocytes containing proteins (transferring receptor) that were no longer required in the mature red blood cell [14], they have subsequently been demonstrated to play a role in activation of the immune response. Exosomes derived from B lymphocytes were able to stimulate CD4+ T cells in an antigen/MHC class II restricted manner [6] and have been demonstrated to be the source of MHC class II molecules on follicular dendritic cells [15]. In addition, tumor cells release vesicles that function in cross-priming by transferring a protein antigen from the tumor cell to a dendritic cell for immune presentation [10,16]. Importantly, dendritic cells release vesicles (named "dexosomes") that have been demonstrated to prime specific T cells *in vitro *and eradicate established murine tumors [5]. In vitro, dexosomes have the capacity to present antigen to naïve CD8+ cytolytic T cells and CD4+ T cells [17,18].

Human dexosomes are enriched in the components necessary to function as an antigen-presenting entity. Extensive electron microscopic and protein characterization has revealed that dexosomes contain a specific set of proteins that differentiate them from other plasma membrane derived vesicles (such as apoptotic cells for example), including MHC class I and II molecules and CD1a, b, c, d molecules, as well as the co-stimulatory molecule CD86 and several tetraspan proteins (CD9, CD37, CD53, CD63, CD81, and CD82) [Anosys unpublished data, [19,20]].

Dexosomes have been demonstrated to participate in antigen presentation in the following way [21,22]. After capturing antigens at the periphery, DC incorporate MHC-antigenic peptide complexes in dexosomes with immunostimulating factors. Released dexosomes subsequently transfer MHC-antigenic peptide complexes and associated proteins to antigen-naïve DC in the regional lymph nodes. The latter thereby acquire the ability to stimulate CD4+ and CD8+ T cells. Thus, dexosomes appear to act as a vehicle for disseminating antigen amongst DC, representing a potentially important mechanism of immune response amplification. This hypothesis forms the rationale for the potential use of dexosomes as a therapeutic cancer immunotherapy.

Dexosomes have demonstrated significant antitumor activity in a mouse tumor model, suggesting that the use of dexosomes derived from dendritic cells may result in improved efficacy relative to the *ex vivo *dendritic cell approach for eradication of advanced cancer. Purified dexosomes were shown to be effective in both suppressing tumor growth and eradicating an established tumor in this model. Furthermore, the effect of the dendritic cell-derived dexosome was greater than that of the dendritic cell from which it was produced [5]. Therefore, we hypothesized that dendritic cell-derived dexosomes would be an effective platform for activating tumor antigen-specific immune responses in humans.

We performed this study to investigate the safety, feasibility, and efficacy of administering autologous dexosomes loaded with tumor antigens (subsequently referred to as DEX) to patients with advanced NSCLC. We also evaluated the immunologic responses in selected patients and monitored the clinical outcomes.

## Methods

### Patients

This phase I clinical protocol was approved by the Duke University Medical Center Institutional Review Board and conducted in compliance with the Helsinki Declaration and under an IND from the United States Food and Drug Administration held by Anosys Corporation. All subjects provided written informed consent. Patients were eligible for enrollment if they had histologically confirmed, unresectable Stage III A or B or Stage IV NSCLC, were HLA A*0201 positive, at least 18 years of age, and had adequate organ function and a Karnofsky performance status of at least 80%. Patients were required to have been treated with at least one prior standard chemotherapy regimen and have measurable disease. In addition, patients were required to have tumor expressing MAGE A3 or MAGE A4. To avoid performing repeat biopsies, this was achieved by detecting MAGE A3 or MAGE A4 expression in peripheral blood tumor cells by RT-PCR using established methods.

The main exclusion criteria were: prior therapy within 4 weeks of the leukapheresis, CNS disease, history of autoimmune disease, concurrent use of systemic steroids, presence of HIV infection or acute or chronic viral hepatitis B or C. Pregnant or lactating women were also excluded.

### Manufacture of DEX

Dexosomes were manufactured from peripheral blood mononuclear cells (PBMCs) as previously described [23]. Briefly, PBMCs were obtained from the patient during a 2-blood volume leukapheresis and shipped overnight to Anosys, Inc., Menlo Park CA. The cells were washed, adhered to plastic to isolate monocytes and placed in a 7-day serum-free culture at 37°C in a humidified 5% CO_2 _atmosphere in the presence of 50 ng/mL GM-CSF (Immunex, Seattle, Washington) and 10 ng/mL of IL-4 (Schering-Plough, Kennilworth, NJ). On the 7^th ^day of culture, the supernatant of the resulting dendritic cell preparation was harvested, filtered, and concentrated. Dexosomes were then isolated by ultracentrifugation on a D_2_O/sucrose cushion. As described in table 1, the final dexosome product (DEX) consisted of one of three different formulations based on different methods for loading the following peptides onto the dexosomes: MAGE-derived, HLA-A2 restricted Class I peptides KVAELVHFL (MAGE-A3(112–120)), GVYDGREHTV (MAGE-A4(230–239)) and GLYDGMEHL (MAGE-A10(254–262)); MAGE derived HLA-DP04 restricted Class II peptide TQHFVQENYLEY (MAGE-A3(247–258)) [24]; and the control peptides, the cytomegalovirus (CMV) pp65-derived, HLA-A2 restricted Class I peptide NLVPMVATV and the tetanus toxoid-derived, promiscuous HLA-DR Class II peptide QYIKANSKFIGITE (produced by Multiple Peptide Systems, San Diego, CA). Peptides were loaded either "directly" onto dexosomes (i.e., following purification of dexosomes from the DC culture) or "indirectly" (i.e. onto cultured DCs that are the source of the dexosomes). The quantity of DEX prepared from a single leukapheresis was measured by ELISA as previously described [23]. The measured number of MHC class II molecules present in the DEX product was utilized for the purpose of dosing. The final DEX product was diluted in 0.9% normal saline for injection, sterile filtered, and stored at -80°C; subsequently the DEX product was shipped overnight to the investigative site, and maintained in its frozen state until 1 hour before use.

**Table 1:** Dose Groups and Product Formulations

Dose Cohorts	Number of patients in Cohort (Patient number)	Peptides loaded/HLA class	Peptide loading method and concentration	DEX dose (expressed as numbers of MHC class II molecules)
A	3 (DU 5, 6, 8)	MAGE-A3 (112–120)/class I MAGE-A4 (230–239)/class I MAGE-A10 (254–262)/class I CMV pp65/class II Tetanus toxoid/class II	Indirect (10 μg/mL) Indirect (10 μg/mL) Indirect (10 μg/mL) Indirect (10 μg/mL) Indirect (10 μg/mL)	0.13 × 10 ^14^

B	5 (DU 24, 39, 44, 50, 63)	MAGE-A3 (112–120)/class I MAGE-A4 (230–239)/class I MAGE-A10 (254–262)/class I CMV pp65/class I MAGE-A3 (247–258)/class II Tetanus toxoid/class II	Direct (10 μg/mL) Direct (10 μg/mL) Direct (10 μg/mL) Direct (10 μg/mL) Indirect (10 μg/mL) Indirect (10 μg/mL)	0.13 × 10 ^14^
C	4 (DU 49, 73, 81, 83)	MAGE-A3 (112–120)/class I MAGE-A4 (230–239)/class I MAGE-A10 (254–262)/class I MAGE-A3 (247–258)/class II	Direct (100 μg/mL) Direct (100 μg/mL) Direct (100 μg/mL) Indirect (10 μg/mL)	0.13 × 10 ^14^

The amino acid sequences of the peptides used for loading the dexosomes are: MAGE-A3(112–120) = KVAELVHFL; MAGE-A4(230–239) = GVYDGREHTV; MAGE-A10(254–262) = GLYDGMEHL; MAGE-A3(247–258) = TQHFVQENYLEY; CMV pp6 = NLVPMVATV; tetanus toxoid = QYIKANSKFIGITE

Note: DU39, DU44, and DU83 were not treated.

### Treatment and Follow-up Schedule

Patients were enrolled into three cohorts (A,B,C) that varied in the method of MHC Class I peptide loading and concentration as described in Table 1. The quantity of DEX administered to the patients in each cohort was identical: 1.3 × 10^13 ^MHC class II molecules in a volume of 3 mL (divided into twoinjections given at two sites on opposite sides of the body) as a combination of subcutaneous (90% of the volume) and intradermal (10%) injections weekly for 4 weeks. No retreatment was allowed. Vital signs were monitored for 1 hour after each injection. Clinical responses were assessed by RECIST criteria. CT scans of the chest through the upper abdomen were obtained at baseline, 1 month following the last dose of DEX and every 3 months after last dose of DEX for 1 year, but scans to confirm responses were not required in this phase I study. All surviving patients have been followed every 6 months for assessment of vital status.

### Delayed type hypersensitivity (DTH) testing

Prior to the initial leukapheresis and 1 week after the last dose of DEX, the following peptides were injected intradermally, in addition to the standard recall antigen panel of Candida, Mumps, and tetanus: MAGE-A3(112–120), MAGE-A4(230–239), MAGE-A10(254–262), and MAGE-A3(247–258), each at 10 μg in 0.1 mL saline. The diameter of the induration and erythema was measured 48 hours following the peptide injection.

### ELISPOT testing

Immune response was evaluated at baseline and 1 week following last dose of DEX with cryopreserved PBMCs obtained by leukapheresis. The ELISPOT assay was performed by ImmunoSite, Inc. (Pittsburgh, PA) according to previously reported methods [25] using both direct assessment of thawed PBMCs, and when possible, following *in vitro *stimulation of PBMCs with autologous DCs pulsed with the MAGE-A3(112–120), MAGE-A4(230–239), and MAGE-A10(254–262). The number of spots (interferon-gamma-secreting T cells) per 20,000 responding PBMC was reported. The background number of spots against an irrelevant antigen was subtracted from the number of spots for the experimental conditions.

### Natural killer cell activity

NK cells were isolated from cryopreserved PBMCs using an NK Cell Isolation Kit (Miltenyi Biotec, Bergisch Gladbach, Germany) according to the manufacturer's instruction. NK cell purity was checked by flow cytometry using anti-CD3-FITC, anti-CD45-PerCP, and anti-CD56-APC antibodies (BD Bioscience, San Jose, CA). Isolated NK cells and NK cells activated for 40 hours by IL-2 (Proleukin, Chiron) 600 Units/ml were incubated at various effector to target rations with chromium-51 labeled K562 cells, an NK target, for 4 hours at 37°C and cytotoxicity was assessed by the amount of radiolabeled chromium released. Cytotoxicity was calculated as follows: percentage of target cell lysis = 100 × (counts per minute (cpm) of experimental release - cpm of spontaneous release) / (cpm of maximum release - cpm of spontaneous release).

### Statistics

The primary endpoints of this study were safety and feasibility, with secondary endpoints of clinical and immunologic response rates. The incidence, type and severity of adverse events were recorded during the study treatment through 30 days following the last dose of DEX. Descriptive statistics were used to present the data. Adverse events were coded using MEDDRA version 5.0. Survival and time to progression were measured from the date of the first injection to the date of documented disease progression or death. For patients who progressed, the time to disease progression was determined by the interval from the first injection+ 1 day to the last evaluation of disease staging. For patients who did not progress or die during the two year follow up period, the time of disease progression and survival was determined by the interval between the first dose of DEX and the date of last evaluation of disease staging + 1 day and concatenated with the '+' sign.

## Results

### Patient Characteristics

Thirteen patients, (8 female, 5 male) median age 62 years (range 44–72 years) with unresectable pretreated Stage III or IV NSCLC were enrolled. The median time from original diagnosis to study entry was 9.9 months (range 2–61 months) and the median Karnofsky score of the patient population was 80% (range 80–100). DEX therapy was administered to 9/13 (6 female and 3 male) patients. Of the 9 dosed patients, 5 patients had Stage IV and 4 patients had Stage IIIB disease. Six patients had stable disease and 3 patients had progressive disease at study entry. Two patients had squamous cell carcinoma, 4 patients had adenocarcinoma, 2 patients had large cell carcinoma, and in 1 case the histological type was not reported. All patients had received prior chemotherapy (median number of cycles: 6.5, range: 3–30), 6/9 patients had received prior radiotherapy and 4/9 patients had prior surgery for cancer treatment. Four patients did not receive DEX for the following reasons: manufacturing failure in 2 cases (DU39, DU83), one of whom had received chemotherapy 13 days prior to leukapheresis and one of whom (DU39) also had rapid disease progression at the time of leukapheresis; delay in shipment in one instance (DU14); and, rapid disease progression prior to planed dosing with DEX in one case (DU44). The characteristics of all dosed patients are listed in Table 2 (see separate file for Table 2).

### Dexosome manufacture

The dose of DEX that was selected corresponded to the maximum dose that could be achieved from healthy donors. We confirmed that this dose could be generated in all but two patients with NSCLC. The mean dexosome generation consisted of a total class II number of 3.14 × 10^14 ^(range 4.1 × 10^12 ^to 9.1 × 10^14^). This quantity of dexosomes in our advanced NSCLC patients is similar in quantity to that generated from healthy donors (mean for 111 healthy donors was 3.9 × 10^14 ^total class II).

### Toxicity

The DEX immunotherapy was generally well tolerated without evidence of serious toxicity. The most frequently reported adverse events causally related to the use of DEX were mild (Grade 1–2) in severity and included: Injection site reactions (erythema, contusion, induration and edema) in 8 patients; flu like syndrome (1 patient); and, peripheral edema and pain in the arm (1 patient). There were no significant organ or laboratory toxicities attributable to the vaccine. No autoimmune reactions were observed.

### Immunologic Response

#### DTH analysis

All 9 dosed patients underwent DTH testing with individual tumor-associated peptides prior to and following all doses of DEX. There was no DTH response to the specific peptide antigens prior to DEX therapy. Three patients (DU06, DU24 and DU49) had a positive response of at least 5 mm erythema or induration in the longest dimension 48 hours after skin testing with one of the MAGE peptides. Specifically, DU06 had 5 mm induration and erythema with MAGE-A4(230–239), DU24 had 6 mm induration and erythema with MAGE-A10(254–262) and DU49 had 5 mm induration and erythema with MAGE-A3(112–120), respectively.

#### In vitro immunologic analysis

The peptide-specific immune response to MAGE and CMV was analyzed using ELISPOT in 5 of 9 dosed patients (DU24, DU49, DU50, DU63, DU81). One patient (DU49) exhibited detectable increases in T cell precursor frequency to MAGE-A10(254–262) following in-vitro stimulation (an increase of 12 MAGE-A10-specific cells/20,000 responders). Assays for DU50 and DU63 could not be completed because of poor viability. Robust responses to anti-CD3 and to the control peptide CMV pp65 were observed in DU24, DU81, and DU49, but no MAGE-specific responses were detected.

Since most patients did not exhibit a significant increase in antigen-specific T cell activity, we hypothesized that regulatory influences such as CD4+CD25+ regulatory T cell populations might inhibit augmentation of the T cell response. In 2/3 patients who had analyzable specimens available, an increase in CD4+CD25+ T cells as a percentage of CD4+ T cells was observed following completion of DEX therapy when compared with baseline values (DU05: increase from 19.49 to 26.64%, DU08: minimal change from 20.39 to 23.42%, DU50: increase from 17.45 to 31.81%). The small number of samples available for this analysis precludes any conclusions but does suggest that CD4+CD25+ T cell analyses should accompany future studies of DEX immunotherapy.

**Figure 1:**
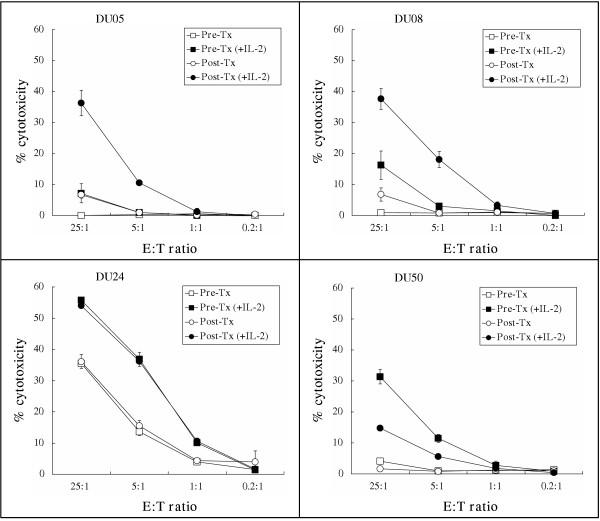
**Cytolytic activity of NK cells. **Cytolytic activity of NK cells isolated from the PBMC of 4 patients (DU05, DU08, DU24, DU50) pre (squares) and post (circles) immunization and cultured with (dark shapes) or without (open shapes) IL-2 was determined. The percentage lysis of the NK target (K562) cells is reported at effector to target ratios of 0.2:1 to 25:1.

During the study, new data from Escudier et al (manuscript submitted) suggested that the immunologic activity of DEX might be due to activation of NK cells. We therefore explored the hypothesis that NK cells may be activated following DEX therapy. This was not planned as part of the initial analysis and therefore specimens of PBMC were limiting in all but 4 patients (DU05, DU08, DU24, DU50). Although there was no consistent change in NK percentage before and after immunization (Table 1: DU05: 10.7 to 9.2%; DU08: 6.0 to 5.4%; DU24: 8.8 to 8.5%; DU50: 9.9 to 13.9%), NK activity as determined by the ability to lyse K562 target cells was observed to increase in 2/4 patients following immunizations (Fig 1 -see additional file Fig 1). Short-term culture with IL-2 was required to activate the NK cells in vitro as there was very low activity in the absence of IL-2. Although addition of IL-2 increased the NK cell activity, it did not change the relative pattern of activity, i.e., in no instance did the order of the results change as a result of IL-2 stimulation.

### Clinical Outcomes

At approximately 2 years of follow up, survival from the first dose of DEX ranged from 52 to 309 days for cohort A, 280 to 665+ days for cohort B, and 244 to 502 days for cohort C (Table 1). In order to obtain preliminary data on response rate, CT scans were obtained prior to immunization and at 1 month and 3 month intervals following completion of the immunizations, but additional scans were not obtained to confirm responses. Of the two patients (DU05, DU08) with disease progression at study entry, DU05 was stable at the end of the immunizations but was felt to have clinically progressed at day 88 shortly before death. DU08 also had stable disease at the end of the immunizations and on every three month follow-up until having progression at day 302. Of four additional patients (DU06, DU24, DU63, DU81) who began the study with stable disease, two (DU24 and DU63) have remained without progression for greater than 12 months. DU06 was stable at the post-immunization CT but subsequently died unexpectedly of unknown etiology and without a follow-up scan, and DU81 was stable at the post-immunization CT but had progressed by the next CT scan at the three month follow-up. The remainder of the patients had progressed at the post-immunization CT scan including DU73 who had disease progression prior to the first dose of DEX. The time until progressive disease, as documented from the first dose of DEX, ranged from 30+ to 302 days for cohort A, 40 to 429+ days for cohort B, and 51 to 166 days for cohort C.

## Discussion

The objective of this study was to show that DEX could be manufactured from NSCLC patients and could be safely administered. We demonstrated the feasibility of producing dexosomes loaded with specific MAGE and other peptides and demonstrated that this form of immunotherapy was well tolerated in patients with advanced NSCLC. Leukapheresis products could be shipped to a central processing facility with good cell viability after transport in a majority of cases, in contrast to other autologous therapies involving tissues where logistics of tissue harvest and processing are complex. The dexosome product was successfully manufactured and loaded with multiple peptides in the majority of patients. This suggests that different panels of tumor antigen-derived peptides could be successfully loaded onto dexosomes. Using multiple peptide panels may allow for targeting various tumor types and larger patient populations, and decreases the likelihood of antigenic escape.

We observed increases in systemic immune responses against MAGE by DTH reactivity in 3/9 patients who had no reactivity to the MAGE peptides prior to immunization and activation of NK cells, but found minimal increases in antigen-specific T cell activity in *in vitro *assays performed circulating PBMCs. Possible explanations include nonoptimized or low-sensitivity assays, inadequate antigen presentation, counter-regulatory mechanisms that dampen immune responses, or the lack of persistence of antigen-specific Tcells in the circulation (i.e., the T cell may have migrated to tumor tissue or lymph nodes). The possible role of negative regulatory mechanisms was suggested by the presence of elevated levels of CD4+CD25+ regulatory T cells following immunization in some patients.

An intriguing immunologic observation was the increase in NK activity following immunization in 2/4 patients analyzed. Although DEX are intended to activate antigen-specific, MHC-restricted T cell responses, it is possible that cytokines released in response to DEX therapy could cause activation of NK cells or that DEX could directly activate NK cells. DEX therapy may stimulate both innate and adaptive arms of the immune response and thereby provide a rationale for maximizing the anti-tumor effect of this approach, even in cases where tumors have lost Class I antigens, a common finding as cancers become more advanced [26]. Indeed, in a phase I study in melanoma patients, DEX loaded with MAGE peptides were well tolerated and associated with both clinical response and increased NK activity (Escudier, manuscript submitted to J. Trans Med).

Despite the small sample size and the fact that 3/9 dosed patients had disease progression at the time of initiation of DEX treatment, we observed prolonged disease stabilization in some patients. Large clinical trials in patients with advanced NSCLC have generally reported median time to progression of 3–5 months in patients with advanced NSCLC treated with systemic chemotherapy regimens [27-30]. The lack of toxicity and interesting clinical and immunologic observations support further investigation of DEX immunotherapy as a treatment approach for both advanced and early stage NSCLC and other tumors. Phase II clinical studies in non-small cell lung cancer and other tumor types are planned to continue to explore the efficacy of this novel immunotherapy.

## Conclusion

DEX therapy was well. Immune activation and stability of disease was observed in some immunized patients with advanced NSCLC.

## Competing Interests

Michael Morse received funding from NIH 5R21CA89957-02. Additionally, portions of this study were funded by Anosys, Inc.

Nancy Valente, Revati Shreeniwas, Mary Ann Sutton, Alain Delcayre, Di-Hwei Hsu, and Jean BernardLe Pecq held stock and were employees in Anosys,

H. Kim Lyerly was a consultant for Anosys, Inc.

## Authors' contributions

MAM was the principal investigator of the study and oversaw all aspects including protocol development, patient management, data collection and analysis, and manuscript preparation.

JG enrolled patients to the study and managed their care and participated in data analysis.

Takuya Osada performed the NK assays and analyzed the data.

SK enrolled patients to the study and managed their care.

AH performed in vitro immunologic assays and analyzed the data.

TMC oversaw the immunologic analyses performed at Duke University and analyzed the data.

NV, RS, and MAS oversaw development of the protocol, data collection and analysis, and manuscript preparation.

AD developed and oversaw the MAGE screening for patient eligibility.

D-H H oversaw portions of the immunologic analysis and data analysis.

J-B L provided scientific direction regarding generation of the dexosomes, protocol development, and data analysis and manuscript preparation

HKL provided consultation on immunologic assay development All authors read and approved the final manuscript.

## Supplementary Material

Additional File 1 **Table 2 (DOC) **presents the remainder of clinical and immunological data from all patients Click here for file
