# Supplementary material for: A phase I study of dexosome immunotherapy in patients with advanced non-small cell lung cancer
Source: J Transl Med. 2005 Feb 21;3:9. doi: 10.1186/1479-5876-3-9 (PMC551593; doi:10.1186/1479-5876-3-9)
Supplement: Additional File 1 — Table 2 (DOC) presents the remainder of clinical and immunological data from all patients [file 1479-5876-3-9-S1.doc]

## Table 2:  Patient Characteristics and Response to DEX Treatment

| Demographic and baseline characteristics | | | | | Clinical response | | Immune response | | | | | |
| --- | --- | --- | --- | --- | --- | --- | --- | --- | --- | --- | --- | --- |
| Pt. ID | Age | Sex | Disease/ Stage at study entry | Prior therapy^a^ | TTP_DEX#1_ days | Survival_DEX#1_ days | DTH^b^ | Elispot | | | CD4/CD25 (%CD4) | NK activity^c^ |
| Cohort A |  |  |  |  |  |  |  |  | | |  |  |
| DU5 | 66 | F | PD / IV | Chemo:1 | NC^@#^ | 99 | Neg | ND | | | ↑ | ↑ |
| DU6 | 55 | M | SD / IV | Chemo:3,XrRT1,Surg:1 | 30+* | 52 | Pos | ND | | | ND | ND |
| DU8 | 54 | M | PD / IIIB | Chemo:2,XrRT1,Surg:3 | NC^@#^ | 309 | Neg | ND | | | ↑ | ↑ |
| Cohort B |  |  |  |  |  |  |  |  | | |  |  |
| DU24 | 72 | F | SD / IIIB | Chemo:1,XrRT1,Surg:1 | 429* | 665** | Pos | Neg | | | ND | Unch |
| DU50 | 62 | M | SD / IV | Chemo:2 | 40 | 280 | Neg | ND | | | ↑ | ↓ |
| DU63 | 62 | F | SD / IIIB | Chemo:1,XRT2 | 419* | 489** | Neg | ND | | | ND | ND |
| Cohort C |  |  |  |  |  |  |  |  | | |  |  |
| DU49 | 44 | F | SD / IV | Chemo:4,anti-EGFR:1, XRT:1,Surg:2 | 51 | 502 | Pos | Pos IVS | | | ND | ND |
| DU73 | 46 | F | PD / IV | Chemo:2,XRT:1 | NC@ | 244 | Neg | ND | | | ND | ND |
| DU81 | 70 | F | SD / IIIB | Chemo:2,XRT:1 | 166 | 349 | Neg | ND | | | ND | ND |
|  |  |  |  |  |  |  |  | |  |  |  |  |

*       Three patients who did not progress are censored at their last disease evaluation date.

**     Two patients who were alive are censored at the last date when known to be alive.

@ DU5, DU8, and DU73 had PD between the time the dexosomes were generated and the initiation of immunization; therefore TTP was not calculated (NC).

^#^ DU5 had stable disease at the first follow-up CT scan (disease for 30 days after 1^st^ immunization) but died of what was thought to be progressive disease before further scans were obtained; DU8 was progressing at study entry, but after 1^st^ immunization had stable disease for 302 days.

Abbreviations:

­ = increase     ↓ = decrease       ND=Not done     IVS= in vitro stimulation       Unch=unchanged      Pos=positive    Neg=negative

TTP_DEX#1_ = time to progression from first DEX injection; Survival_DEX#1_ = time to death from first DEX injection

SD = stable disease; PD = progressive disease

^a^ Chemo = chemotherapy, XRT = x-ray therapy, Surg = surgery (each followed by number of treatments)

   EGFR = epidermal growth factor receptor; anti-EGFR is a monoclonal antibody treatment (see patient DU49)

^b^ Positive is defined as at least 5 mm in the maximum dimension of erythema or induration 48 hours after injection of any of the Class I peptides (MAGE A3, A4, A10) and class II (Mage A3 DPO4).

^c^ NK cells activated by IL-2 for 40 hrs
